# Supplementary material for: A new class of binding-protein dependent solute transporter exemplified by the TAXI-GltS system from Bordetella pertussis
Source: Commun Biol. 2025 Aug 12;8:1201. doi: 10.1038/s42003-025-08591-x (PMC12344293; doi:10.1038/s42003-025-08591-x)
Supplement: Supplementary file 1 — Supplementary information [file 42003_2025_8591_MOESM1_ESM.pdf]

Supplementary Information

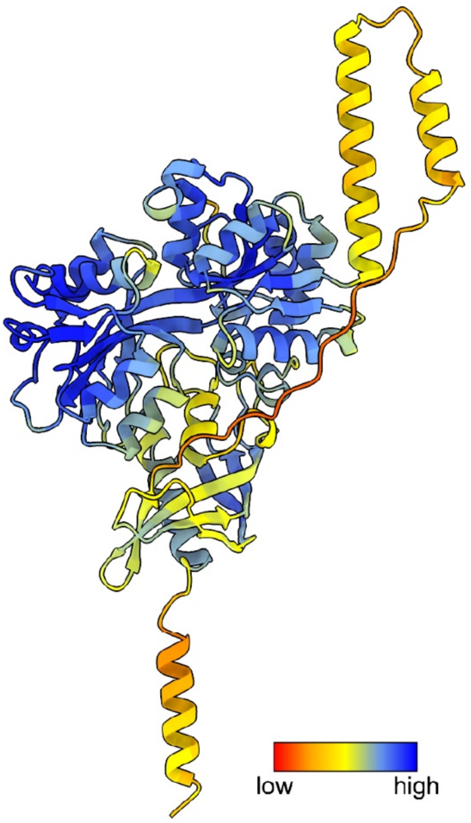

Supplementary Figure 1. Predicted local distance difference test (pLDDT) score for BP0403. AlphaFold 2 model of BP0403 (AF-Q7VSK8-F1) coloured by pLDDT score.

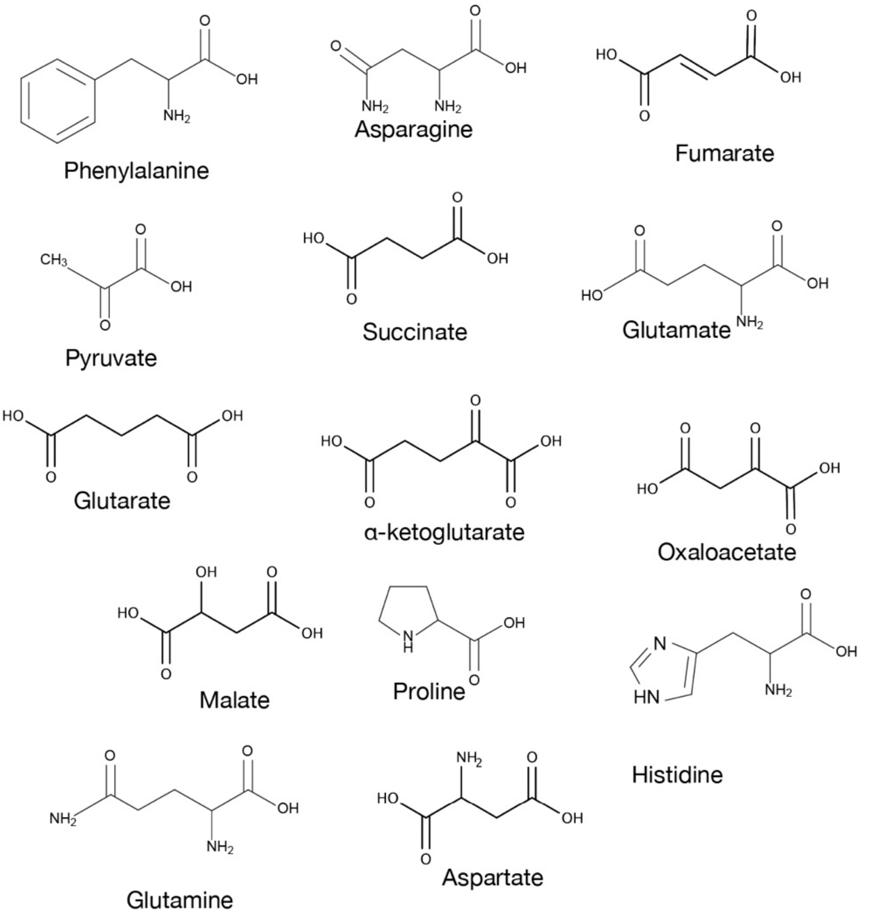

Supplementary Figure 2. Chemical structures of potential ligands used in the DSF ligand screen of BP0403.

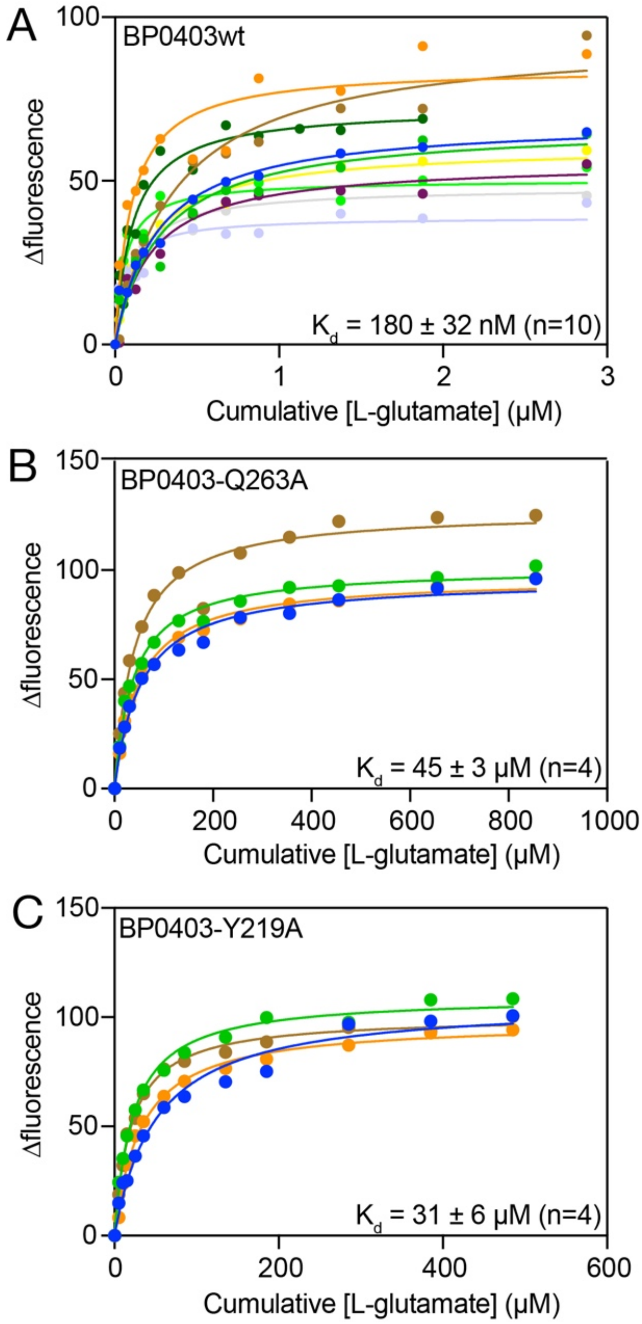

Supplementary Figure 3. L-glutamate binding curves. Binding curves derived from tyrosine fluorescence titrations for **A)** BP0403wt, **B)** BP0403-Q263A and **C)** BP0403-Y219A. Average Kd for each data set is shown in bottom left corner. Error represents S.E.M. and n is indicated. Each individual data set is represented by a different color.

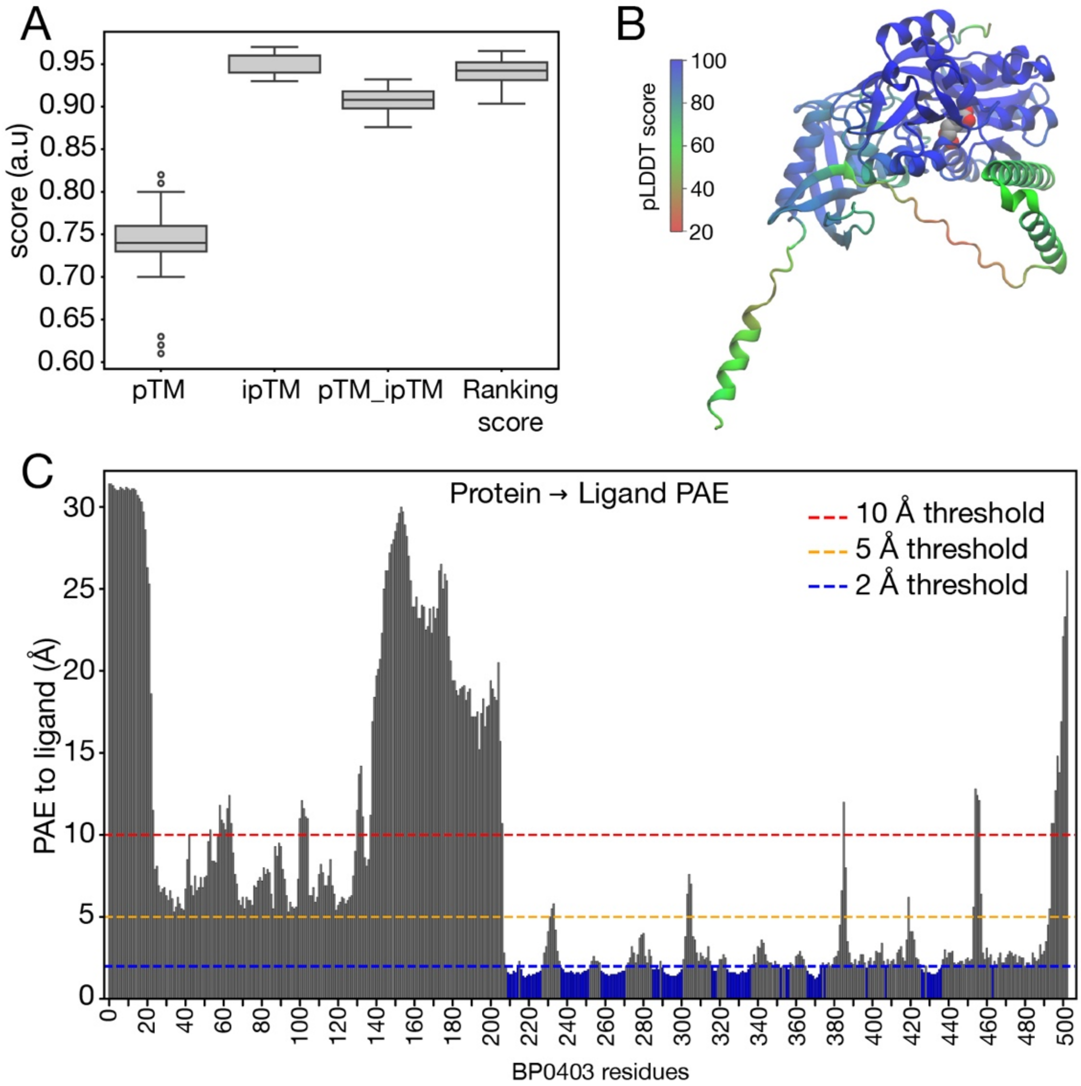

Supplementary Figure 4. L-glutamate bound BP0403 monomer structural model. A) pTM, ipTM, combined pTM_ipTM, and Ranking score of the 250 BP0403 monomer AF3 predictions. **B)** Highest ranking score model coloured by pLDDT. **C)** PAE values of the best model, calculated in the direction from BP0403 residues to L-glutamate atoms. Lower values reflect higher confidence in the predicted relative positions, assuming the protein is correctly aligned. Asymmetry is expected due to the small size and flexibility of the ligand.

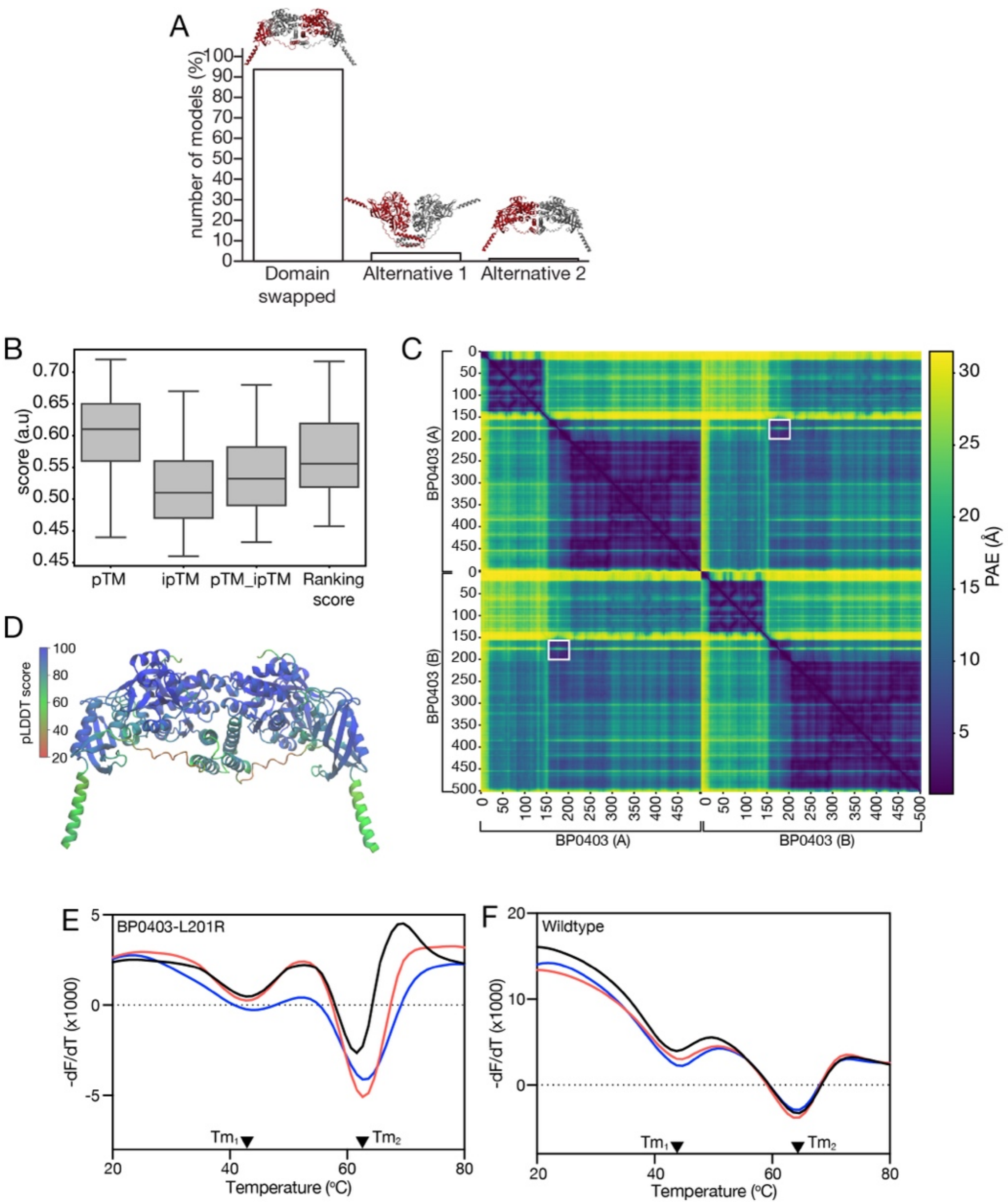

Supplementary Figure 5. BP0403 dimer structural model and DSF analysis of BP0403 monomerization mutant (BP0403-L201R). A) Three observed arrangements in the BP0403 dimer models; domain-swapped and 2 alternatives. Three different distances were measured (A94-A415', A94-A423, D399-K400'), and the sampled arrangements were binned based on them. **B)** pTM, ipTM, combined pTM_ipTM, and Ranking score of the 1500 BP0403 dimer AF3 predictions. **C)** Best model in terms of the Ranking score coloured by pLDDT. **D)** PAE matrix of the best model. The portion of HD/HD' helices interactions is highlighted with a white box. **E-F)** Derivatives of the unfolding curves (dF/dT) for **E)** BP0403-L201R and **F)** wildtype BP0403. 3 technical replicates are shown for each and the location of the 2 minima (Tm1 and Tm2) are indicated.

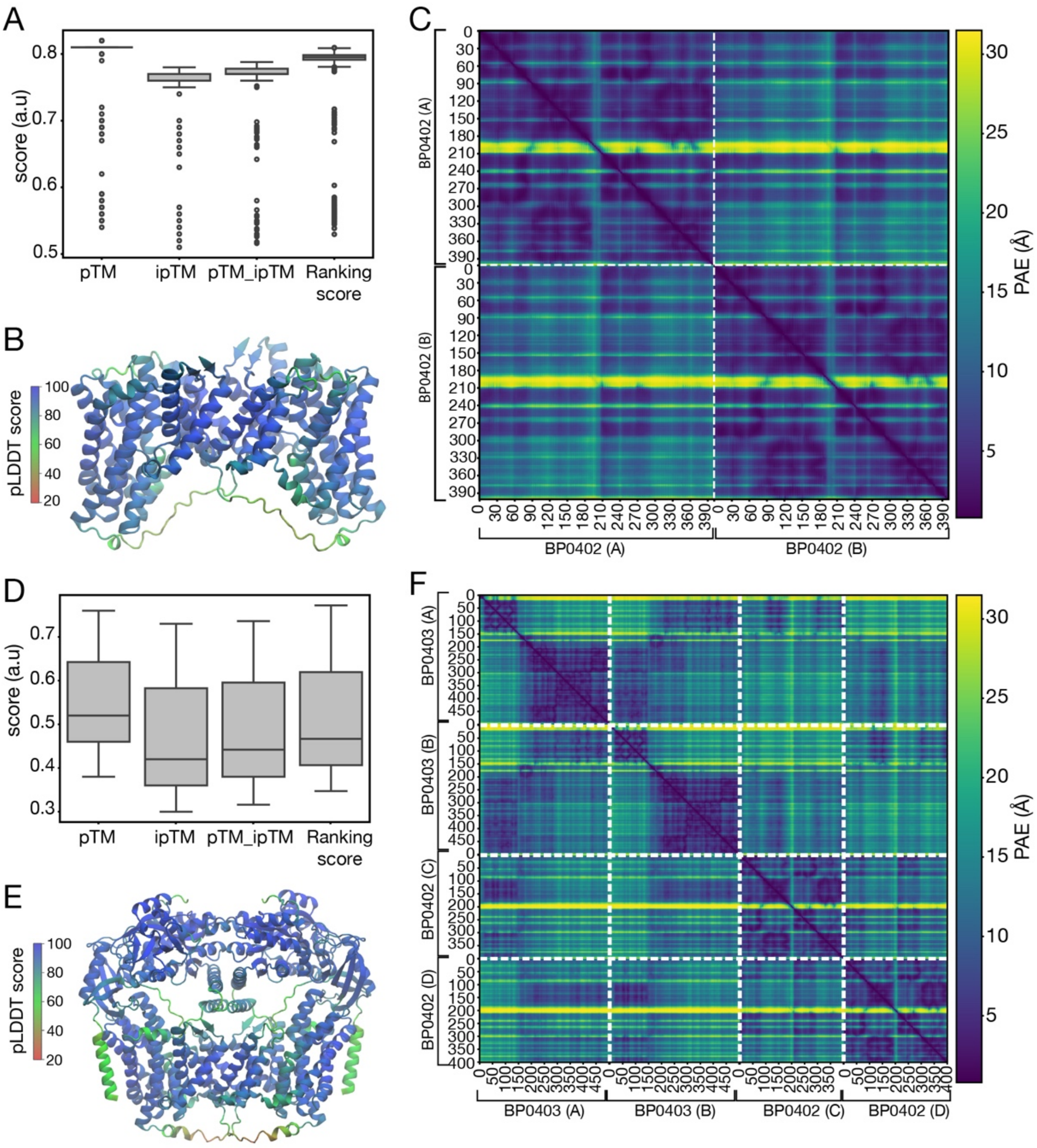

Supplementary Figure 6. GltS dimeric and BP0403:GltS heterotetrameric models. A) pTM, ipTM, combined pTM_ipTM, and Ranking score of the 5000 GltS dimer AF3 predictions. **B)** Best GltS dimer prediction in terms of the Ranking score coloured by pLDDT. **C)** PAE matrix of the best GltS dimer model. **D)** pTM, ipTM, combined pTM_ipTM, and Ranking score of the 1500 BP0403:GltS heterotetramer AF3 predictions. **E)** Best BP0403:GltS heterotetramer coloured by pLDDT. **F)** PAE matrix of the best BP0403:GltS heterotetramer prediction.

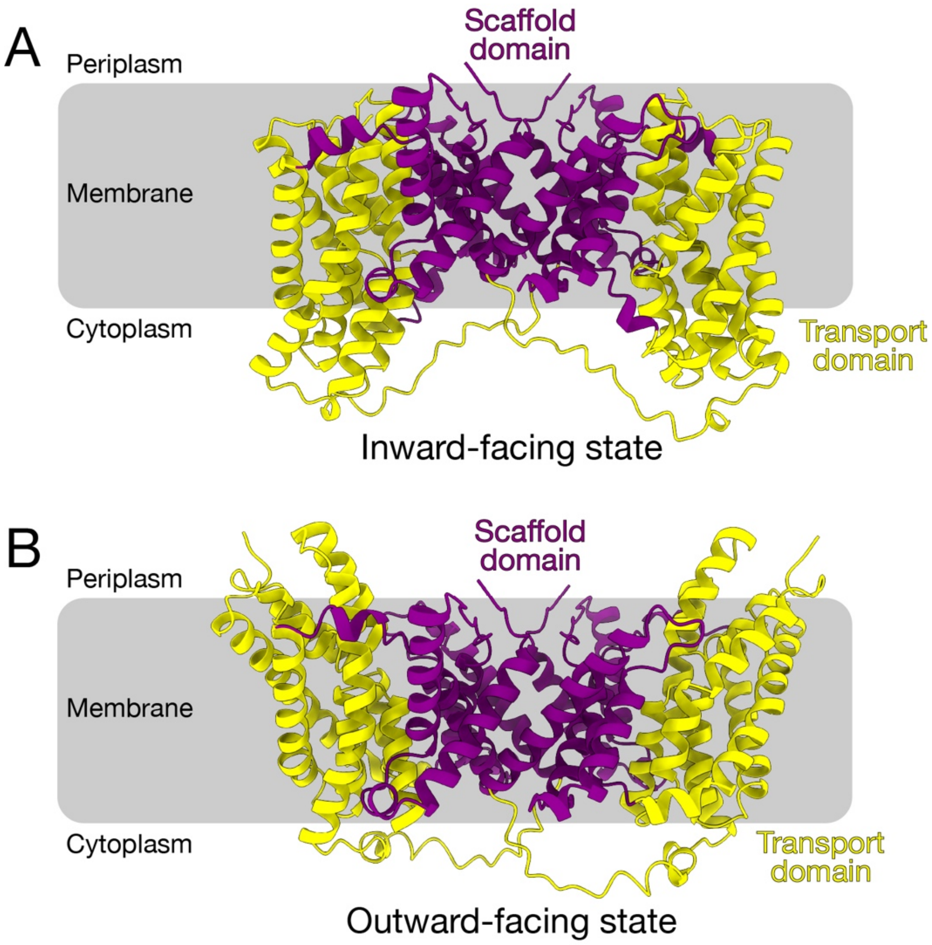

Supplementary Figure 7. Comparison of GltS AlphaFold 3 models. A) The GltS dimer modelled alone is depicted in the inward-facing state with the substrate binding site on the cytoplasmic side of the membrane. **B)** GltS outward-facing state model depicted in the BP0403:GltS heterotetrameric model. For each model the scaffold domain is coloured purple and the mobile transport domain is coloured yellow.

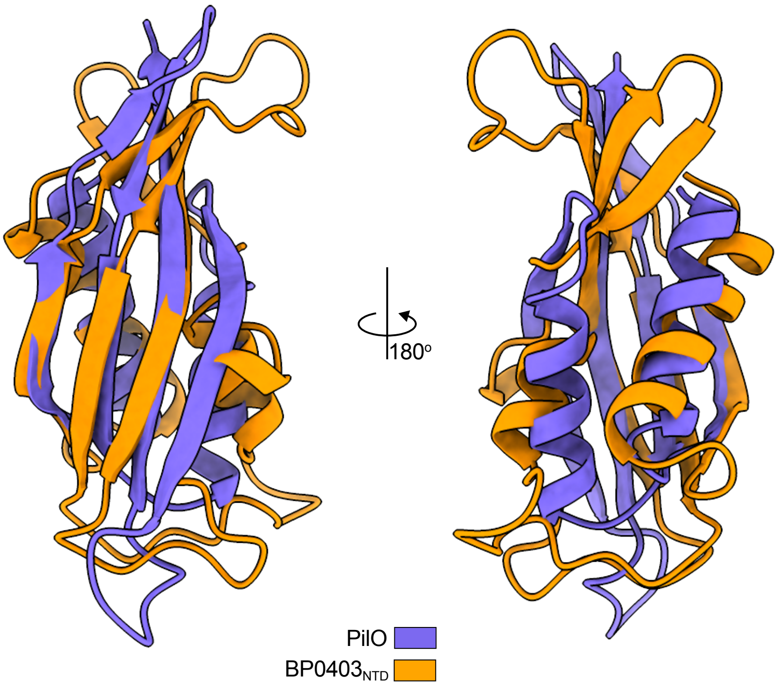

Supplementary Figure 8. Structural comparison of N-terminal domain of BP0403 with PilO. Superimposition of $BP0403_{NTD}$ model (orange structure) with the X-ray crystal structure of PilO from *Pseudomonas aeruginosa* (PDB ID: 2RJZ, purple structure, Sampaleanu et al. doi.org/10.1016/j.jmb.2009.09.037).
