# Supplementary material for: A new class of binding-protein dependent solute transporter exemplified by the TAXI-GltS system from Bordetella pertussis
Source: Commun Biol. 2025 Aug 12;8:1201. doi: 10.1038/s42003-025-08591-x (PMC12344293; doi:10.1038/s42003-025-08591-x)
Supplement: Supplementary file 3 — Description of Additional Supplementary Materials [file 42003_2025_8591_MOESM3_ESM.pdf]

Description of Additional Supplementary Files

File name: Supplementary Data 1

Description: The source data for all graphical data
